# Cone-Beam Angle Dependency of 3D Models Computed from Cone-Beam CT Images

**DOI:** 10.3390/s22031253

**Published:** 2022-02-07

**Authors:** Myung Hye Cho, Mohamed A. A. Hegazy, Min Hyoung Cho, Soo Yeol Lee

**Affiliations:** 1 R&D Center, Ray, Seongnam-si 13494, Korea; myunghye.cho@raymedical.co.kr (M.H.C.); hegazy@khu.ac.kr (M.A.A.H.); 2 Department of Biomedical Engineering, Kyung Hee University, Yongin-si 17104, Korea; mhcho@khu.ac.kr

**Keywords:** cone-beam dental CT, cone-beam artifact, digital impression, half-scan image reconstruction, stereolithography

## Abstract

Cone-beam dental CT can provide high-precision 3D images of the teeth and surrounding bones. From the 3D CT images, 3D models, also called digital impressions, can be computed for CAD/CAM-based fabrication of dental restorations or orthodontic devices. However, the cone-beam angle-dependent artifacts, mostly caused by the incompleteness of the projection data acquired in the circular cone-beam scan geometry, can induce significant errors in the 3D models. Using a micro-CT, we acquired CT projection data of plaster cast models at several different cone-beam angles, and we investigated the dependency of the model errors on the cone-beam angle in comparison with the reference models obtained from the optical scanning of the plaster models. For the 3D CT image reconstruction, we used the conventional Feldkamp algorithm and the combined half-scan image reconstruction algorithm to investigate the dependency of the model errors on the image reconstruction algorithm. We analyzed the mean of positive deviations and the mean of negative deviations of the surface points on the CT-image-derived 3D models from the reference model, and we compared them between the two image reconstruction algorithms. It has been found that the model error increases as the cone-beam angle increases in both algorithms. However, the model errors are smaller in the combined half-scan image reconstruction when the cone-beam angle is as large as 10 degrees.

## 1. Introduction

Most dental computed tomography (CT) systems are based on the circular cone-beam scan geometry, and they use two-dimensional flat panel X-ray detectors (FPDs) to detect X-rays. The circular cone-beam scan geometry not only simplifies the scan mechanics, but also makes it possible to realize a three-dimensional (3D) CT with less cost than is necessary for a conventional medical CT based on the helical scan. The Feldkamp reconstruction algorithm [1] is mostly used to reconstruct 3D images for a dental CT since it is computationally efficient. However, the Feldkamp algorithm produces cone-beam artifacts that become more visible as the vertical cone-beam angle increases. If the projection data are acquired in the circular cone-beam scan geometry, cone-beam artifacts are inevitable since the projection data set is mathematically incomplete to solve the image reconstruction problem [2]. The cone-beam artifacts appear in the form of conical shadings or streaks around high-contrast objects in the sagittal- or coronal-view images while they appear in the form of circular intensity anomalies in the axial-view images [3].

In modern digital dentistry [4,5], precise 3D digital models, often called digital impressions, of the teeth and the surrounding bones are required for computer-aided design (CAD) and computer-aided manufacturing (CAM) of dental restorations or orthodontic devices [6,7]. Optical intraoral scans can provide a 3D digital model without the need to make physical elastomeric impressions, thus reducing manufacturing and delivery time and costs [8,9]. However, optical intraoral scans are often prone to errors when the tooth surface has non-uniform optical reflectivity [10]. Once a physical impression or a plaster cast model has been made, an optical scan of the plaster model or an X-ray CT scan of the impression or the plaster model may provide more precise 3D digital models than an intraoral optical scan does. Extraoral optical scans of a plaster model are more frequently used than X-ray CT scans because of the superior spatial resolution of 3D optical scanners. However, on the other side, both intraoral and extraoral optical scanners have limitations in scanning hidden structures between teeth such as narrow openings or cracks [11,12]. Therefore, an X-ray CT scan of a physical impression is now of great interest since it can provide images of the hidden and internal structures as well [13]. An X-ray CT scan of a physical impression has another advantage over an optical scan in that a plaster model is also prone to errors caused by the volume change during their solidification.

There were several reports on the accuracy comparison of the 3D models generated from an optical scanner and a CT scanner. Kulczyk et al. compared the accuracy of the 3D models of a single tooth generated from an optical scanner, a cone-beam medical CT, and a micro-CT. They concluded that a high-resolution CT can provide 3D models whose accuracy is comparable to those made by an optical scanner [11]. Fahrnia et al. compared the 3D models of a human skull generated from an optical scanner and a CT. They reported that the CT-based skull models have bigger errors in some parts of the skull as compared to the optical-based model without specifying the source of this error [14]. Da Silva-Dantas et al. [15] compared the accuracy of linear measurements in the optical-based and cone-beam CT-based 3D models of plaster models. They reported that the cone-beam CT and the optical scanner showed similar performance in the linear measurement, but in some plaster models, there was a small degree of distortion in the CT-based 3D models [15]. Yousefi et al. [16] and Kim et al. [17] also had a similar study and reported that the CT-based model had slightly larger errors than the optical-based model.

There were a couple of reports on the use of a micro-CT to make digital models of an extracted tooth for the morphological [13,18] or compositional analysis [19]. Recently, a successful digital model generation from the micro-CT images of physical impressions has been reported [20]. As dental CT technology develops, it becomes more feasible to make a digital impression directly from the dental CT images of a patient without making a physical impression or a plaster model. Recently, it was reported that a precise digital impression can be made from the micro-CT images of a full-arch physical impression and the accuracy of the 3D digital impression is better than that made from the intraoral optical scan [20]. In this work, the CT images of the physical impression were taken with setting the vertical cone-beam angle to near zero to mitigate the cone-beam artifacts. However, if a digital impression is to be computed from the clinical CT images, the vertical cone-beam angle could be big enough to induce cone-beam artifacts, depending on how the X-ray detector is positioned against the X-ray source in the dental CT machine and how the patient is positioned inside the scanner.

Cone-beam artifacts can be reduced by applying ray-angle-dependent weighting in the backprojection with the cost of extensive computation [21,22]. Mori et al. introduced an efficient cone-beam artifact reduction method that exploits the cone-beam artifact characteristic of appearing unequally in the circular scan direction in the half-scan (HS) reconstructed images [3]. In the HS image reconstruction, the projection data within a scan angle of π + 2γ_m_ is used, in which γ_m_ is the horizontal cone-beam angle, whereas the whole projection data over 2π is used in the full-scan (FS) image reconstruction. Since cone-beam artifacts appear less severely in a small angular range in the HS reconstructed images and the angular range rotates as the HS range rotates, cone-beam artifacts can be reduced in the overall angular range by selectively taking the less severe regions from the multiple HS reconstructed images and combining them.

To investigate the cone-beam angle dependency of 3D model errors, we computed 3D stereolithography (STL) data from the 3D micro-CT images of plaster models taken at three different cone-beam angles, and we compared them with the reference STL data obtained from optical scanning of the plaster models. We used the FS Feldkamp (FDK) algorithm [1] and the combination-weighted HS algorithm (CW-FDK) [3] for the CT image reconstruction, and we analyzed the 3D model errors for the two image reconstruction algorithms.

## 2. Methods

### 2.1. Acquisition of Projection Data Using a Micro-CT

**Figure 1:**
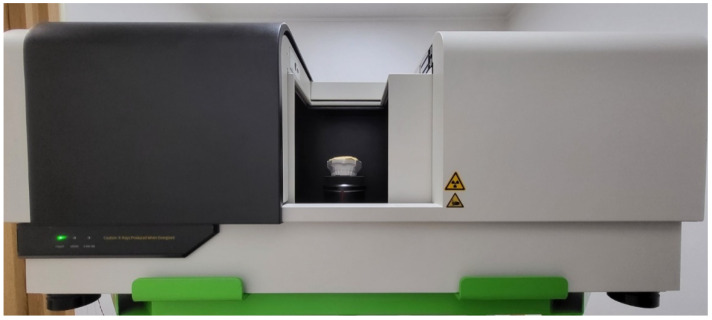
The micro-CT used for the experiment. The rotating stage can be moved vertically to adjust the cone-beam angle of the ray passing through the center of the object.

For the CT scan, we used a micro-CT consisting of a flat-panel detector (FPD) with a matrix size of 1256 × 1256 and an X-ray source with a focal spot size of 40 µm. The FPD has a CsI scintillator layer to convert X-ray photons to visible photons and its pixel pitch is 119 µm. The X-ray tube voltage and current are controllable in the range of 50–80 kV and 0.4–0.7 mA, respectively. The micro-CT has a rotating stage for the cone-beam scan in between the X-ray source and the FPD as shown in Figure 1. The magnification ratio of the micro-CT is fixed at 1.7:1 and the nominal spatial resolution of the CT images is 68 µm. The field-of-view (FOV) of the micro-CT scan is 8.5 cm × 8.7 cm, and the reconstructed 3D image has the isotropic voxel size of 68 μm.

**Figure 2:**
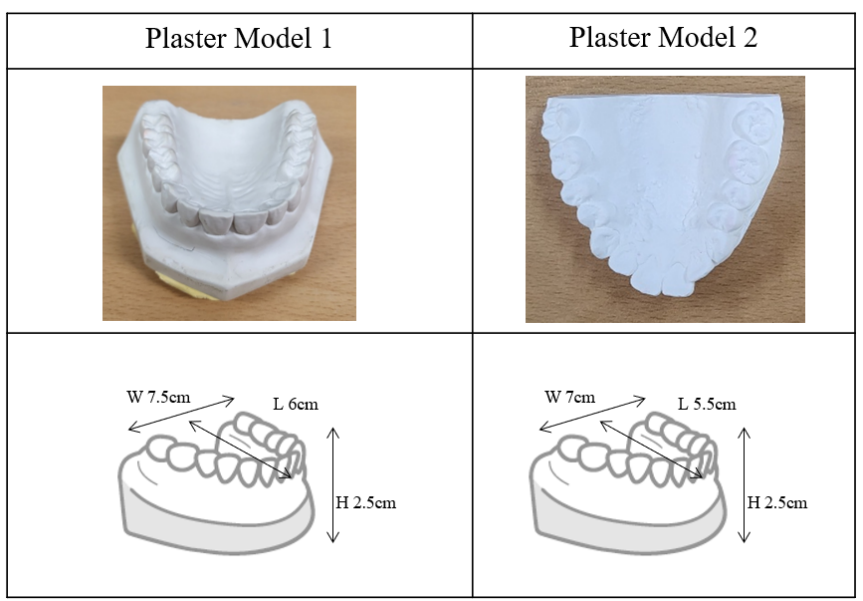
The photographs and dimensions of the plaster models used for the micro-CT scan.

**Figure 3:**
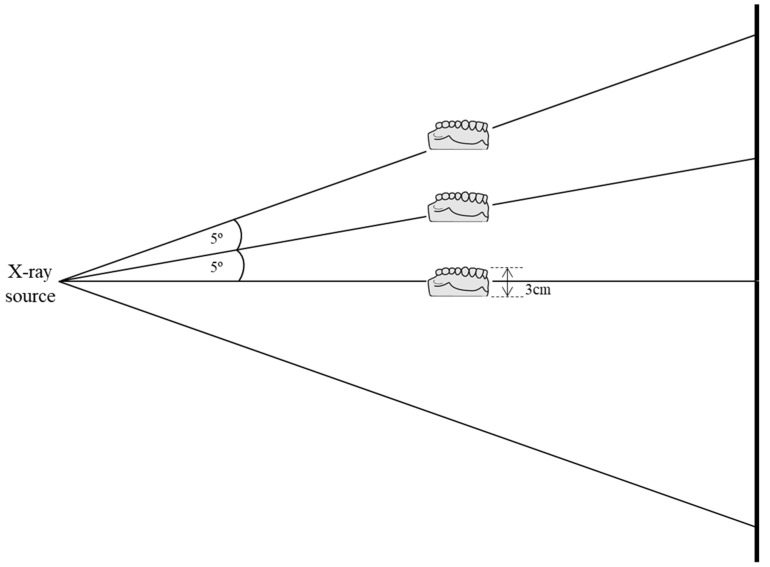
The cone-beam angles adopted in the micro-CT scan. The cone-beam angle is the vertical angle of the ray passing through the center of the object.

We took the projection data of the plaster cast models taken from volunteers, shown in Figure 2, with the tube voltage and current of 80 kV and 0.7 mA, respectively. The number of projection views was 720 which resulted in the scan time of 72 s for each scan with the detector frame time set to 100 ms. We performed full scans of a scan object over the projection view angle of 2π. After finishing a full scan at a given vertical cone-beam angle, we changed the vertical position of the object to make a different vertical cone-beam angle. The vertical cone-beam angle was defined by the vertical ray angle of passing through the center of the object’s middle plane. For measuring the vertical angle, we used a projection image, i.e., a radiographic image, taken at the starting angle of the scan. After measuring the vertical height of the middle plane on the projection image, we computed the vertical angle with the known source-to-detector distance of 34.0 cm and source-to-object distance of 19.8 cm. We performed three full-scans for each object with the cone-beam angles of 0, 5, and 10 degrees as shown in Figure 3.

### 2.2. CT Image Reconstruction

**Figure 4:**
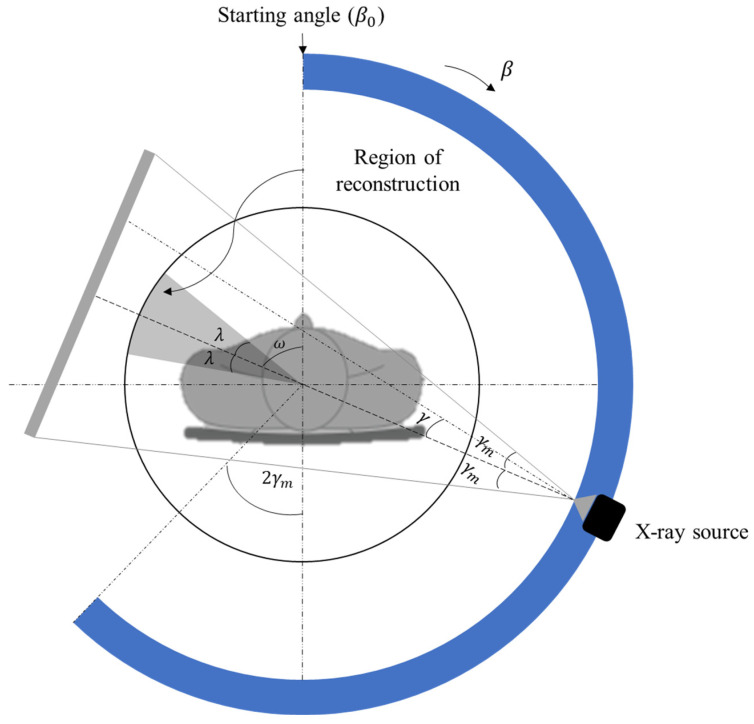
The half-scan geometry seen on the central plane. The half-scan range is from the starting angle (β_0_) to the end of the blue arc. The region of reconstruction for this half-scan range is the shaded fan of an angular span of 2λ.

We used the two popular image reconstruction methods, one full-scan (FS) Feldkamp algorithm (FDK) and the other half-scan (HS) Feldkamp algorithm. In contrast to a full scan over 2π on the circular scan trajectory, a half scan is performed over the limited angular range of π + 2γ_m_ in which 2γ_m_ is the horizontal cone-beam angle as indicated in Figure 4. Since the scan time of a half scan is shorter than that of a full scan, a half scan has an advantage over a full scan when motion artifacts are concerned. However, HS images have lower SNR than FS images due to the partial use of the projection data in the image reconstruction.

Unlike the FS image reconstruction in which every projection data is equally weighted in the filtered backprojection, a proper weighting must be applied to the projection data in the HS image reconstruction to take into account the double angular sampling at some projection view angles [23]. Otherwise, the double angular sampling effects may appear as shading artifacts in the HS reconstructed image. We applied the following weighting function to the projection data prior to the filtered backprojection in the HS image reconstruction [3]:

(1) $$w\left(\gamma ,\beta \right)=\{\begin{array}{ll} \sin^{2}\left(\frac{\pi }{4}\frac{\beta }{\gamma_{m}-\gamma }\right), & \;\beta \in \left[0,2\gamma -2\gamma_{m}\right]\\ 1, & \;\beta \in \left[2\gamma -2\gamma_{m},\pi -2\gamma \right]\;\\ \sin^{2}(\frac{\pi }{4}\frac{\pi +2\gamma_{m}-\beta }{\gamma_{m}+\gamma }), & \;\beta \in \left[\pi -2\gamma ,\pi +2\gamma_{m}\right] \end{array}$$

where β is the projection view angle on the circular scan trajectory, γ is the horizontal ray angle inside the cone, and γ_m_ is the angular span of the cone in the horizontal direction as shown in Figure 4. The weighting function not only takes account of the double angular sampling, but also mitigates the truncation effects caused by the half-scan.

It is known that cone-beam artifacts in HS images are least appearing at the opposite side, around the angle of ω, to the center of the HS view-angle range [β_0_, β_0_ + π + 2γ_m_] in which β_0_ is the starting angle of the half-scan as shown in Figure 4 [3,22]. To reduce cone-beam artifacts, it is reasonable to reconstruct a partial image only at the fan-shaped (wedge-shaped in the 3D images) sub-region of the angular span of 2λ as indicated in Figure 4. Setting the sub-region size, i.e., λ, is a matter of trade-off between cone-beam artifacts and computation time. To obtain a complete image over the full region, we need to reconstruct partial images by rotating the starting angle by 2λ until we can make a full image by combining the partial images. If we set λ to π/*N_BP_*, we can make a seamless full image, *I_full_*, by combining the partial images of *P_i_* (*i* = 1, 2, …, *N_BP_*):

(2) $$I_{full}=\sum_{i=1}^{N_{BP}}P_{i}.$$

For each FS projection data acquired from the micro-CT, we reconstructed 20 HS images in the respective sub-region, and we combined them to make a complete image. We also reconstructed a full-scan image at each cone-beam angle using the FS Feldkamp algorithm for the sake of comparison. For the sake of shorter notation, we will use FDK for the FS Feldkamp algorithm and CW-FDK for the combination-weighted HS Feldkamp algorithm.

In addition to cone-beam artifacts, beam hardening artifacts may also have negative effects on the computation of a digital impression. Due to the polychromatic nature of an X-ray beam, the projection data does not exactly represent the line integral of the X-ray attenuation coefficients inside the scanned object, which may result in shading and streak artifacts in the reconstructed image. We applied the beam hardening correction (BHC) to the projection data before the image reconstruction. In the BHC, we multiplied the projection data by the conversion factors that have been obtained by polynomial fitting of the measured attenuation to a function of the line integral length.

### 2.3. STL Data Generation from the Micro-CT Images

**Figure 5:**
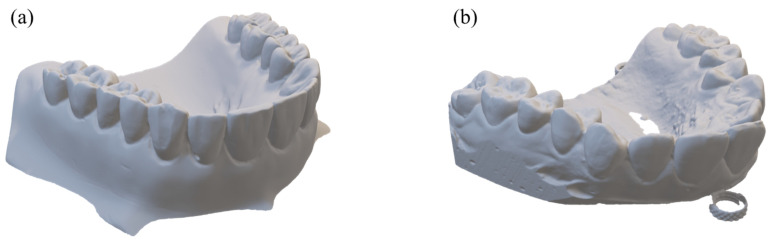
The plaster models, (**a**) Model 1 and (**b**) Model 2, in the STL format obtained from the optical scanning.

To generate STL data from the CT images of a plaster model, we need to segment the plaster parts from the 3D CT images. From the CT-number histogram of all the pixels in the CT images taken at zero cone-beam angle, we found the two peaks corresponding to the air and the plaster. We found an optimal global threshold around the middle of the two peaks by a manual search so that the STL data generated from the CT images best match to the reference STL data obtained from the optical scanning of the plaster model. We applied the global threshold to all the CT images acquired at different cone-beam angles. For the STL data generation from the CT images, we used the STL generation tool, Raydent Studio (Ray, Korea). After generating the STL data for the whole region of the plaster model, we manually cropped the region of interest covering the teeth and bones. For the optical scanning to generate the reference STL data, we used the 3D optical scanner (Identica Hybrid, 3D Wib, Korea). Figure 5 shows the two plaster models in the STL format obtained from the optical scanning. These two STL data will be used as references when the STL data computed from the CT images are evaluated.

To compare the reference STL data with other STL data obtained from the CT images, we used the reverse engineering tool, Geomagic Control X (Artec 3D, Luxembourg). With the SW tool, we computed the deviation maps on which the Euclidean distance of the surfaces on the CT-image-derived model from the surface on the reference model are displayed.

## 3. Results

**Figure 6:**
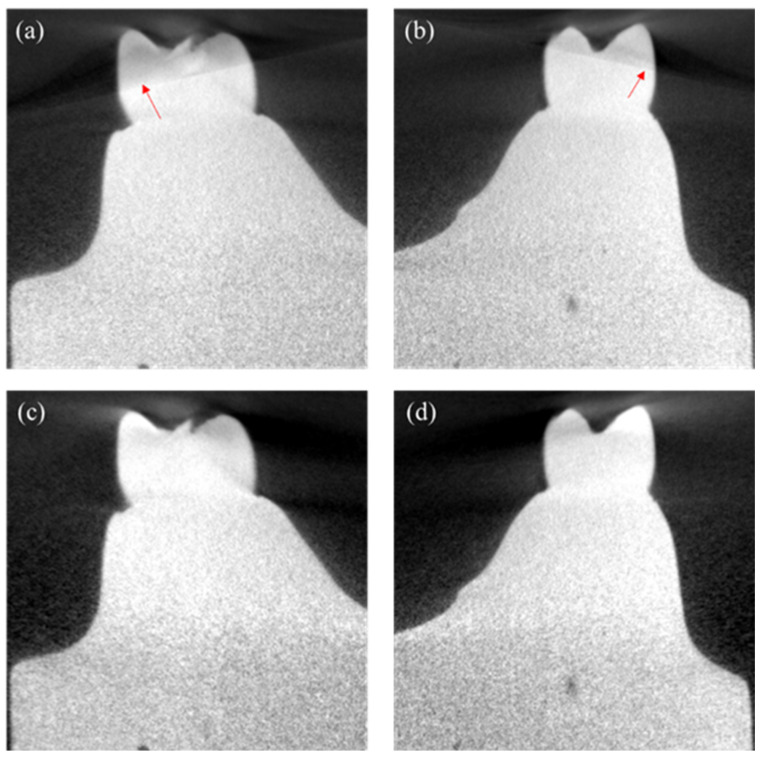
(**a**,**b**) show two regions of interest in the images reconstructed by the FDK algorithm. (**c**,**d**) show the corresponding regions in the images reconstructed by the CW-FDK algorithm. We can see that the cone-beam artifacts (indicated by the red arrow) have been much reduced in the images of the CW-FDK algorithm.

We took CT images of the two plaster cast models at three different cone-beam angles, 0, 5, and 10 degrees. In Figure 6, we have shown zoomed ROIs from the sagittal images of Model 1 taken at 10 degrees. At this large cone-beam angle, we can see strong cone-beam artifacts appearing in the form of shadings and streaks as indicated by the arrows. The cone-beam artifacts are not visible in the images taken at the small cone-beam angle of 0 or 5 degrees.

**Figure 7:**
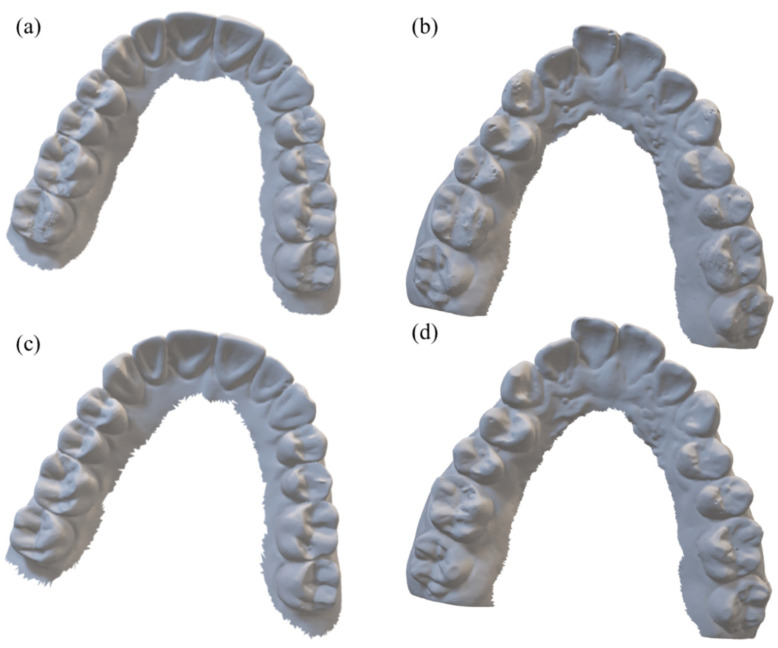
The 3D models computed from the CT images of (**a**,**c**) Model 1 and (**b**,**d**) Model 2 taken at the cone-beam angle of 10 degrees. The top and bottom rows have been obtained from the images reconstructed by FDK and CW-FDK, respectively.

Figure 7 shows the 3D model images computed from the CT images taken at the cone-beam angle of 10 degrees. The first row shows the images of Model 1 and Model 2 computed from the FS images and the second row shows the corresponding images computed from the combined HS images. By visual inspection of the 3D model images, it is difficult to detect the difference even though we can clearly see the difference in the two-dimensional CT images shown in Figure 6.

**Figure 8:**
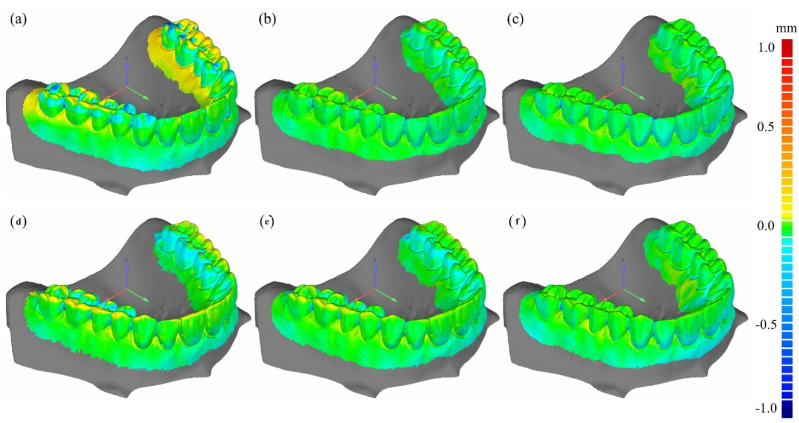
The deviation maps of Model 1 as compared to the optical STL (reference). (**a**–**c**) show deviation maps computed from the FDK images taken at the cone-beam angles of 10, 5, and 0 degrees, respectively, while (**d**–**f**) show deviation maps computed from the CW-FDK images taken at the cone-beam angles of 10, 5, and 0 degrees, respectively.

**Figure 9:**
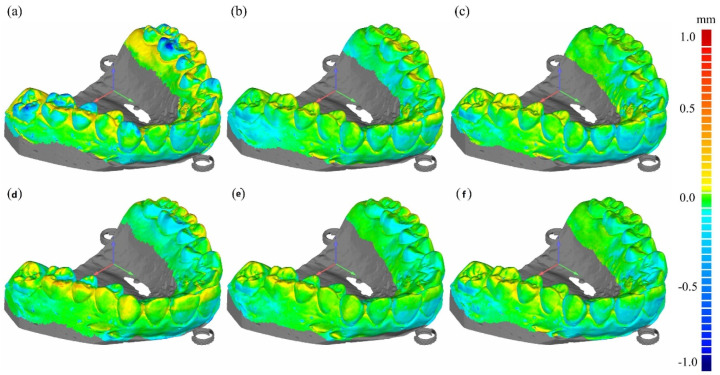
The deviation maps of Model 2 as compared to the optical STL (reference). (**a**–**c**) show deviation maps computed from the FDK images taken at the cone-beam angles of 10, 5, and 0 degrees, respectively, while (**d**–**f**) show deviation maps computed from the CW-FDK images taken at the cone-beam angles of 10, 5, and 0 degrees, respectively.

Figure 8 shows the deviations, from the reference 3D model, of the 3D models computed from the CT images of Model 1 taken at the cone-beam angles of 0, 5, and 10 degrees. The deviation at a surface point on the 3D model represents the Euclidean distance from the corresponding surface point on the reference 3D model. The deviation maps at the top and bottom rows are the cases for the FS images and the combined HS images, respectively. Even though it was difficult to visually detect the difference from the 3D model images shown in Figure 7, the difference is now clearly visible in color codes. We can notice that the pair of two deviation maps, computed from the FDK and CW-FDK images taken at 0 degrees, have little difference between them. However, in the cases of 5 and 10 degrees, there are noticeable differences, particularly around the molar tooth regions. Figure 9 shows the deviation maps for the 3D models computed from the CT images of Model 2 taken at the cone-beam angles of 0, 5, and 10 degrees. We can see similar deviation behaviors in this case too.

**Figure 10:**
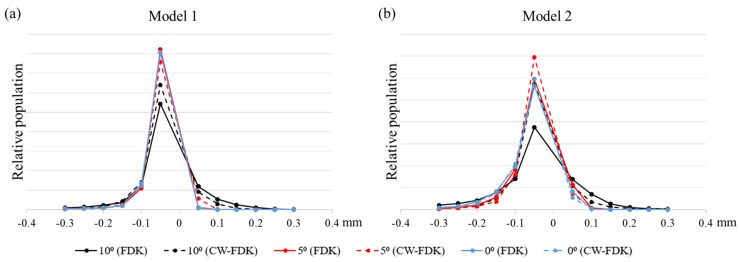
The relative population of the deviations from the reference of (**a**) Model 1 and (**b**) Model 2 at three different cone-beam angles of 0, 5, and 10 degrees. The solid and dashed lines correspond to the cases of the FDK and CW-FDK algorithms.

Figure 10 shows the relative histograms of the deviations in the case of Model 1 (left) and Model 2 (right). The histogram shows the normalized population of the Euclidean distances from the reference 3D model at all surface points. The population of surface points on a model depends on the shape of the model and the type of the image reconstruction, and it ranges from 53,000 to 140,000. At the cone-beam angles of 0 and 5 degrees, there is little difference between the cases of FDK and CW-FDK, meaning that the cone-beam artifacts have little effect on the 3D model generation. However, at the cone-beam angle of 10 degrees, the histograms are widened due to the increased deviations at more surface points. However, the widening is less in the case of CW-FDK, which means CW-FDK is better than FDK in terms of the 3D model error.

Table 1 summarizes the mean of the positive deviations (MPD) and the negative deviations (MND) at all surface points of the 3D models. The positive and negative deviations mean bigger and smaller sized models than the reference model, and those two mean values matter in the fabrication of dental restorations or orthodontic devices. In both cases of Model 1 and 2, FDK and CW-FDK show similar model errors at the low cone-beam angles of 0 and 5 degrees, but CW-FDK shows smaller model errors at the large cone-beam angle of 10 degrees.

## 4. Discussion

The 3D model computed from the micro-CT images may deviate from the reference model and the deviation increases as the cone-beam angle increases. The image reconstruction algorithms also have effects on the deviation. In this study, we used the two image reconstruction algorithms which are computationally efficient. The FDK algorithm is known to be superior to the CW-FDK algorithm in terms of the image SNR. For a given pixel in the 3D CT images, the number of rays used for CW-FDK is less than that for FDK by a factor of (π + 2γ_m_)/2π. Therefore, the image SNR in the CW-FDK images is lowered approximately by a factor of $1/\sqrt{2}$ if 2γ_m_ << π. The lowered SNR can have negative effects on the 3D model computation. It has been observed that residual beam hardening artifacts after the beam hardening correction (BHC) are less in the FDK images. It seems that the conjugate rays coming from the opposite direction somehow lessen the beam hardening effects on the FDK images. In CW-FDK, there is no use of conjugate rays. At the low cone-beam angles, the 3D model errors are similar between FDK and CW-FDK, which suggests that the lower performance of CW-FDK in image SNR and BHC is somehow balanced by the reduced cone-beam artifacts. However, when the cone-beam angle is as large as 10 degrees, the negative effects of the cone-beam artifacts on the FDK images can outweigh the gain in the image SNR and BHC. Therefore, the CW-FDK algorithm can be a good option when the 3D model is generated from the CT images taken at a large cone-beam angle.

In dental CT machines capable of the panorama scan, it is often the case that the X-ray beam is pointing upward to keep the cervical spine bones from interfering with the panoramic image of the teeth and their surrounding bones. It means that the cone-beam angle of the central plane is always non-zero; therefore, there are always some cone-beam artifacts around the teeth. Therefore, when cone-beam artifacts appear in the CT images reconstructed by the FDK algorithm, the CW-FDK algorithm can be used for generating the 3D model.

Computation time is a great concern in dental CT image reconstruction because of the large 3D image matrix size. Since most of the computation time in the image reconstruction is spent on the backprojection, the number of backprojections is the most influential factor that determines the computation time. In FDK, the number of backprojections is linearly proportional to the number of ray projections for a given image matrix size. In the present study, the number of ray projections for a given pixel was 720 for the FDK image reconstruction, and 484 for the CW-FDK image reconstruction. The CW-FDK image reconstruction needs extra computations for the HS weightings, but their computation time is much shorter than that of the backprojections. For a 3D CT image with a matrix size of 1250 × 1250 × 1280, when reconstructed from a projection data with a matrix size of 1256 × 1256 × 720, the computation time of the CW-FDK image reconstruction was 66 s which was 7% shorter than the FDK image reconstruction time (72 s) when the image reconstruction was performed on a desktop computer (Xeon W-2123 @ 3.60 GHz, Intel, Santa Clara, CA, USA) having a 4608-core GPU (TITAN RTX, NVIDIA, Santa Clara, CA, USA) and 32 GB of RAM.

Apart from cone-beam artifacts, there are many other types of artifacts in dental CT images such as metal artifacts [24,25,26], motion artifacts [27,28,29], and limited-view-induced streak artifacts [30,31,32]. These artifacts can also induce errors in the 3D models. However, there has been big progress in reducing the artifacts and noises in CT images by using deep learning techniques [33,34]. The low spatial resolution of a clinical dental CT machine is another factor that will have effects on the 3D model error. The spatial resolution of clinical dental CT machines would be lower than that of the micro-CT used at the present study at least by a factor of 2. Therefore, we need to have more studies, based on the clinical dental CT images having all kinds of artifacts, to further evaluate the feasibility of generating 3D models directly from the dental CT images.

## 5. Conclusions

Cone-beam artifacts can induce significant model errors when the 3D model of an object is computed from the 3D cone-beam CT images of the object. When the cone-beam angle is small, the FDK algorithm has better performance than the CW-FDK algorithm in terms of the model error. However, the situation is reversed when the cone-beam angle is large. Therefore, cone-beam artifacts should be considered in generating 3D models from the CT images taken at a large cone-beam angle. Since the present study is based on the micro-CT images of plaster models, more studies with clinical dental CT images are needed to evaluate the clinical applicability of the 3D models generated directly from clinical dental CT images.

## Figures and Tables

**Table 1:** The mean of positive deviations (MPD) and the mean of negative deviations (MND) in the 3D models.

Cone-Beam Angle (Degrees)	Model 1 MPD/MND [mm]		Model 2 MPD/MND [mm]	
	FDK	CW-FDK	FDK	CW-FDK
10	0.0644/−0.0928	0.0414/−0.0667	0.0736/−0.1343	0.0634/−0.0697
5	0.0184/−0.0506	0.0263/−0.0540	0.0384/−0.0666	0.0310/−0.0530
0	0.0180/−0.0512	0.0185/−0.0497	0.0346/−0.0751	0.0313/−0.0721
